# Translation, validation and test–retest reliability of the VISA-G patient-reported outcome tool into Danish (VISA-G.DK)

**DOI:** 10.7717/peerj.8724

**Published:** 2020-03-04

**Authors:** Jens Erik Jorgensen, Angela M. Fearon, Carsten M. Mølgaard, Jens Kristinsson, Jane Andreasen

**Affiliations:** 1 Sofiendal Aalborg Sundhedsteam, Physiotherapy Private Practice, Aalborg, Denmark; 2 University of Canberra Hospital, Research Institute for Sport and Exercise, Faculty of Health University of Canberra, Bruce, ACT, Australia; 3 Aalborg University Hospital, Department of Physiotherapy and Occupational Therapy, Aalborg, Denmark; 4 Aalborg University Hospital, Department of Orthopaedic Surgery, Aalborg, Denmark; 5 Public Health and Epidemiology Group, Aalborg University, The Faculty of Medicine, Department of Health Science and Technology, Aalborg, Denmark

**Keywords:** VISA-G.DK, Hip pain, Lateral, Musculskeletal, Pain, Hip function

## Abstract

The Victorian Institute of Sport Assessment (VISA) questionnaire model is based on item response theory using a graded response (responses reflect increasing difficulty). The purpose of the VISA-G is to monitor patient outcomes and evaluate treatment strategies for people with greater trochanteric pain syndrome (GTPS). The primary aim of the current study was to translate and culturally adapt the VISA-G into a Danish context (DK) through forward and back translation and cognitive interviews. The second aim was to establish test–retest reliability and face validity of the VISA-G into a Danish context (DK). No major disagreements were observed between the original and translated versions of the questionnaire. A total of 58 heterogenous asymptomatic, and 49 symptomatic respondents (response rate: 92% and 78% respectively) completed the VISA-G.DK twice, 1 week apart. The VISA-G.DK had excellent internal consistency (Cronbach’s alpha: asymptomatic = 0.86; symptomatic = 0.98). The test–retest reliability was excellent for the total score: ICC: 0.961 (95% CI [0.933–0.978]). Standard Error Measurement was calculated to be 0.6. Bland–Altman plots showed no significant or relevant differences from test to retest in the total score with mean differences below 1 (0.61). The minimal detectable change was 3.17 for both groups. The VISA-G.DK was found to be valid, reliable and acceptable for use in the Danish population.

## Background

Greater trochanteric pain syndrome (GTPS), previously known as trochanteric bursitis, is relatively common, affecting between 10% and 25% of the older population (Segal et al., 2007; Lievense et al., 2005). GTPS negatively affects sleep quality, physical activity, work participation and overall quality of life (Fearon et al., 2014a); it can be hard to treat effectively (Lequesne et al., 2008; Bird et al., 2001). A recent study found the prevalence of GTPS in Denmark to be 2.9 per 1,000 patients, with patients having a mean age of 50.8 years, and consistent with other studies, women making up 73% of the participants. Due to the impact that GTPS has on patients and its prevalence, it is appropriate to facilitate the use of an appropriate patient-reported outcome measure (PROM) to evaluate the effectiveness of treatment of GTPS in a Danish setting (Riel et al., 2019).

The expression GTPS has been coined to recognise several disorders around the lateral hip that may produce lateral hip pain: including trochanteric bursitis, and, tendinopathy and tears of the gluteus medius and minimus tendons (Lequesne et al., 2008; Connell et al., 2003). Two or more of these pathologies often co-existing (Lequesne et al., 2008; Connell et al., 2003). The aetiology of GTPS is not fully understood, and likely relates to myofascial pain rather than solely inflammation (Segal et al., 2007; Fearon et al., 2014b). Patient’s typically present with pain on weight-bearing and reproducible tenderness in the region of the greater trochanter, buttock, or lateral thigh. While people with GTPS generally respond to some extent to conservative measures (Barratt, Brookes & Newson, 2017; Mellor et al., 2018; Ganderton et al., 2016; Furia, Rompe & Maffulli, 2009; Rompe et al., 2009), it can be challenging to treat (Lequesne et al., 2008; Bird et al., 2001) and surgical intervention may be necessary in obstinate cases (Lustenberger et al., 2011). A recent SR and meta-analysis noted the need for further high-quality studies to determine the most effective treatment for GTPS (Barratt, Brookes & Newson, 2017). High-quality studies need reliable and valid measurement tools.

Despite the prevalence of GTPS, the VISA-G, a condition-specific PROM tool has only been published recently (Fearon et al., 2015). Previously surrogate tools have been used to evaluate the severity of the condition, for example the modified Harris Hip (mHHS) developed for hip osteoarthritis, and the Oswestry disability index, developed for assessing back pain (Reid, 2016). However, the mHHS and the Oswestry disability index have been shown to measure different domains than the VISA-G (Lustenberger et al., 2011) emphasising the need for linguistically and culturally appropriate condition-specific tools to be developed.

Translated versions of the appropriated developed and tested tools are needed when intending to collect data from respondents with culturally and linguistically diverse backgrounds from the index population. Researchers should ensure rigorous cultural adaptation and translation occurs, confirming the equivalent constructs with a comparable metric (Tsang, Royse & Terkawi, 2017).

The purpose of the VISA-G is to monitor patient outcomes and evaluate treatment strategies for people with GTPS (REF). The VISA-G was constructed and tested with the intention that it should be used as a whole, although segments of it may provide clinicians with insight into how their clients are progressing. The VISA-G is based on the Victorian Institute of Sport Assessment (VISA) questionnaire model, which, intern, is based on item response theory, using graded responses (responses reflect increasing difficulty) (An & Yung, 2014). The VISA questionnaire models are valid for the assessment of other lower limb tendinopathies (Robinson Cook et al., 2001; Cacchio, De Paulis & Maffulli, 2014; Hernandez-Sanchez, Hidalgo & Gomez, 2014), and across multiple languages and cultures (VISA-P (Maffulli et al., 2008a; Korakakis, Patsiaouras & Malliaropoulos, 2014; Zwerver, Kramer & Van Den Akker-Scheek, 2009), VISA-A (Lohrer & Nauck, 2009; Maffulli et al., 2008b; Iversen et al., 2016) and VISA-H (Cacchio, De Paulis & Maffulli, 2014)). The The VISA-G questionnaire (Fearon et al., 2015) is a product of a rigorous development process using the COSMIN recommendations. The English version has good internal and intra-rater reliability, and good content and construct validity; concurrent criterion validity has been demonstrated (Fearon et al., 2015).

The purpose of the current study was to translate the VISA-G from English into the Danish language (VISA-G.DK), to conduct a cross-cultural adaptation into a Danish context, assessing validity in the Danish context, and to evaluate the VISA-G.DK reliability.

## Materials and Methods

### Ethical approval

The Ethics committee for North Jutland, Denmark, stated that no approval was necessary as the study did not include an intervention. The study was approved by the Danish Data Protection Agency (J.nr. 2018-42-4655). All participants provided written informed consent. The study complied with the Declaration of Helsinki.

### Translation procedure for the VISA-G questionnaire

The current authors have previously described the study design and methods, which follow best practice guidelines (Jorgensen et al., 2016a; Jorgensen et al., 2016b; Beaton et al., 2000; Wild et al., 2005).

In brief:

1. Permission. The developer of the VISA-G was approached to collaborate on the translation process (Fearon et al., 2015).

2. Forward translation—data acquisition. Two independent bilingual native Danish speakers and residents (T1 and T2), translated the questionnaires into Danish. T1 was a physiotherapist aware of the concepts being examined, whereas T2 had no medical training. The translation was overseen by a third bilingual researcher (JA).

3. Forward translation—Reconciliation. Separate meetings were held between the third researcher and each of the translators (T1 and T2) to address discrepancies in the forward translations. Issues identified in the translation process were identified (Beaton et al., 2000) with any unresolved queries resolved after consultation with the developer (Wild et al., 2005).

4. Back translation—data acquisition. The agreed-upon forward-translated versions of the Danish questionnaire was then translated back into English by two native English speakers resident in Denmark and fluent in Danish (BT1 and BT2). BT1 was a physiotherapist, whereas BT2 had no medical training. BT1 and BT2 were both blinded to the purpose of the questionnaire and had not seen the original VISA-G questionnaire (Beaton et al., 2000).

5. Back translation—Reconciliation. The translated versions were compared to the original version to ensure conceptual equivalence; the remaining discrepancies and ambiguities were resolved between the project managers (JA) (JEJ), BT1 and BT2.

6. Harmonisation. The harmonisation group consisted of the back- and forward translators, a research physiotherapist (CM) and a bilingual competent physiotherapist (JEJ). The project manager (JA) communicated with each member of the harmonisation group individually by email due to geographic differences. Subsequently, through group-email correspondence, the team agreed upon the harmonised version of the Danish VISA-G of the VISA-G.DK questionnaire. All members of the harmonisation group approved the translated versions.

7. Cultural validation. Cognitive interviews of five patients with a diagnose of GTPS, and four physiotherapists with relevant clinical experience were conducted to assess the harmonised version of the VISA-G.DK. A research physiotherapist trained in cognitive interviewing (CM) conducted these interviews at the Department of Orthopaedic Surgery, Aalborg University Hospital. A maximum of 24 h before the interview, each participant completed the VISA-G.DK. The participant was then interviewed to establish how they had interpreted the items. This process provided the opportunity to identify problem questions, minimising future response errors and nonresponse (Beaton et al., 2000).

The interview method employed was ‘concurrent probing’ with the duration varying between 10 and 35 min. During the interview, the participants were asked to verbalise their thoughts and opinions while completing the questionnaire.

8. Cultural validation—Harmonisation. The results from the cognitive interviews were reviewed, and a final translated version of each questionnaire prepared. Issues of interpretation of the questionnaire were documented (Wild et al., 2005; Kuusela & Paul, 2000).

9. Final Translation. The version was were proofread and checked for spelling and grammar errors. The layout was then finalized by the project manager and three research physiotherapists (Beaton et al., 2000).

10. Documentation. The final report documenting the translation procedure was finalised.

### The VISA-G.DK questionnaire

The VISA-G.DK consists of eight questions (Appendix 1). The first question relates to hip pain in general and is scored on a scale from 0 to 10. The remaining seven questions relate to everyday activities and participation (International Classification of Functioning, Disability and Health (ICF)). The activities and participation questions measure the participants ability to lie on the sore hip, walk on stairs, walk on ramps or slopes, move from sitting to walking, work about the house or garden, participate in regular exercise, and weightbearing related pain and function. As with existing VISA scores, the first six questions were scored between 0 and 10, while questions 7 and 8 are scored out of 10 and 30 respectively. Question 8 is divided into three subsections of which only one section is required to be answered. The final score is calculated out of 100 (Appendix 2). The higher the score the less the disability perceived by the individual.

### Participant recruitment

A sample of convenience was recruited at a physiotherapy private practice in Aalborg, Denmark between June 2018 and May 2019.

Following screening for exclusion criteria: current or history of low back or hip pain requiring them to seek treatment for that condition, the VISA-G.DK was sent to 63 asymptomatic participants, of whom 58 completed the online questionnaire twice, 1 week apart (response rate, 92%). The asymptomatic group was heterogeneous, and included respondents’ with no signs or symptoms of GTPS. The symptomatic group included 63 symptomatic patients presenting symptoms of hip pain in weight bearing and the inability to lie on the affected side, of whom 49 completed the online questionnaire twice, 1 week apart (response rate, 78%).

Nonrespondents in both groups were prompted by a reminder e-mail 24 h after the deadline for submission. Respondents failing to respond a second time were excluded (Table 1).

**Table 1:** Respondents characteristics.

	Asymptomatic	Symptomatic
Respondents included (response rate %)	58 (92)	49 (78)
Age mean (±SD)	50 (±8.9)	56 (±10.2)
Gender/sex, *n* (%)	41 women, 17 men (71% women, 29% men)	47 women, 2 men (96% women, 4% men)
Time to complete questionnaire Round 1 Minutes: seconds range (average)	1:20–8:24 (±4:38)	2:36–9:56 (±4:45)
Time to complete questionnaire Round 2 Minutes: seconds range (average)	1:20–7:01 (±3:36)	2:12–8:38 (±3:49)

The the online questionnaire was completed using Survey Monkey (SurveyMonkey Inc.). The security is deemed to be adequate for collecting nonsensitive personal information (sex and age).

### Score calculation

Scores were totalled as per the scoring sheet (Appendix 2). If a respondent answers all three sections of question 8—the lowest activity scenario was scored (Section A), as per recommended by the developer and in keeping with assuming a worst case scenario.

### Cognitive interviews

Face to face cognitive interviews of five patients with GTPS and four experienced clinical physiotherapists were performed to assess the harmonised version of the VISA-G.DK. The patient’s GTPS diagnosis was confirmed as per Ganderton and colleagues (Ganderton et al., 2017). The interviews were, recorded in writing according to the matrix suggested by Conrad & Blair (1996) (Table 2).

**Table 2:** Matrix of recorded interviews in writing.

	Response stage		
Problem type	Understanding	Retrieval/decision	Response formatting
Lexical		X (question 1)	X (question 8)
Inclusion/exclusion			
Temporal	X (question 2)		X (question 8)
Logical			X (question 8)
Computational			

### Statistics

Based on an expected reliability of 0.90, assuming a power of 0.80 and a significance level of 0.05, we calculated that a total sample size of 49 symptomatic and asymptomoatic respondents within the target age-group, would be required for test–retest reliability (Walter, Eliasziw & Donner, 1998; Polit, 2014). To account for non-respondents, we aimed to recruit 63 asymptomatic and 63 symptomatic participants (Polit, 2014).

Internal consistency was determined via Cronbach’s alpha with 0 indicating no internal consistency and 1 corresponding to perfect internal consistency. A two-way random effects model (2.1), with single measures and absolute agreement ICC, was used to express reliability (Tsang, Royse & Terkawi, 2017; SurveyMonkey Inc.). As a coefficient of stability, Pearson’s product moment correlation coefficient (Pearson’s r) was calculated with a two-tailed test of significance. A larger coefficient value indicating stronger test–retest reliability (Tsang, Royse & Terkawi, 2017; Draugalis & Plaza, 2009).

The standard error of measurement (SEM) was calculated by first creating a variable for the difference between the total VISA-G.DK score obtained during the first and the second round (test score—retest score = Difference). We then calculated the standard deviation (SD) and the SEM of the Difference in the VISA-G.DK (SD_Difference_), as suggested by the COSMIN guidelines (Polit, 2014; Terwee et al., 2018).

The mean (SD) and SEM difference were calculated for the test–retest results, as per COSMIN guidelines (Polit, 2014; Terwee et al., 2018).

To interpret the reliability of the change in scores of the VISA-DK, the minimal detectable change (MDC), representing the amount of score change beyond measurement error, was calculated. The MDC represents a score ±1.96 times the SD_Difference_ in the test–retest scores of the VISA-G.DK (Polit, 2014).

Floor and ceiling effects were explored by assessing the distribution of total scores and determining if more than 15% of the participants achieved either the lowest or the highest score (McHorney & Tarlov, 1995).

Bland–Altman plots were used to assess agreement and heteroscedasticity (Bland & Altman, 1986).

Statistics were performed by SPSS Statistics for Mac, version 24 (SPSS Inc., Chicago, IL, USA).

## Results

### Translation

No major problems were observed in the forward translations of the questionnaires and only minor discrepancies were discussed in the harmonizing process, primarily consisting of differences in the choice of synonyms and use of prepositions. Examples are: ‘Question 1: My usual hip pain is…/The current pain in my hip is….…’ ‘Question 2: I can lie on my sore hip……/I can lie on my painful hip’ ‘Question 4: Walking up or down a ramp or slope…./Walking up and down a ramp or incline’. After discussion and reaching consensus on the most suitable wording the discrepancies were corrected.

### Cognitive interviews

The results from the interview with the five patients with GTPS, and four physiotherapists prompted no major changes in the questionnaire; only a few minor changes were made. In question 1 the Danish word ‘sædvanlige’ was used to describe ‘usual pain’. The patients had difficulty in deciding if the pain score related to the actual pain while answering the questionnaire, or the question referred to their usual pain during the day. This will be solved by making a point of this in the instruction manual and pointing out that the question relates to the usual pain during the day. We also added the word ‘gennemsnitlige’, meaning average to the original term of ‘sædvanlige’.

Question 8 repeatedly posed problems in choosing which section to answer. It was found confusing with three sections with almost the same wording. To clarify this section, we have included a clarification question to each section:

Original version question 8, Section A:

‘Section A: My hip pain is so severe that it will stop me from walking, shopping, running or other weight-bearing exercise. If this is so, how much of this activity do you do each day?’

Clarification question 8, Section A:

Section A: Can you answer yes to the following question: ‘My hip pain is so severe that it will stop me from walking, shopping, running or other weight-bearing exercise. If this is so, how much of this activity do you do each day?’ then answer Section A. If you cannot answer yes, proceed to Section B or C.

This clarification was repeated in Section B and C. The question is prioritised in the instruction manual, making it clear to the tester to be sure that the patient understands the construct of question 8 before answering it.

### Additional amendments

In the original VISA-G time intervals in question 2 are ‘for longer than 1 h’, ‘for 30 min to 1 h’, ‘for 15–30 min’, for 5–15 min’. This has been changed in the Danish version to: ‘for longer than 60 min’, ‘for 30–59 min’, ‘for 15–29 min’, ‘for 5–14 min’, as this is in line with the time intervals in question 8 Section A, B and C in the original VISA-G questionnaire.

### Total score

The score in the asymptomatic respondent population (VISA-G.DK *n* = 58) ranged from 86 to 100, out of a possible 100 points (mean = 98.00 and SD ± 4.05), retest (mean = 98.03 and SD ± 4.05).

The score in the symptomatic respondent population (VISA-G.DK *n* = 49) ranged from 48 to 77, of a possible 100 points (mean = 61.94 and SD ± 5.78), retest (mean = 61.94 and SD ± 5.78).

### Validation and reliability

The asymptomatic respondent population Cronbach’s Alpha showed excellent internal consistency (0.86). The test–retest reliability was excellent: ICC: 0.98 (95% CI [0.97–0.99]). There was a positive correlation between the test and retest variables, *n* = 58, *r* = 0.98, *p* = 0.01.

The symptomatic respondent population Cronbach’s Alpha showed excellent internal consistency (0.98). The test–retest reliability was excellent: ICC: 0.96 (95% CI [0.93–0.98]). There was a positive correlation between the test and retest variables, *n* = 49, *r* = 0.96, *p* = 0.01.

MDC was found to be ±3.17. SEM was calculated to be 0.6.

No floor or ceiling effects were identified in the patient group with less than 15% of participants scoring the minimum or maximum values. In the symptomatic group, VISA-G.DK data were normally distributed. A total of 94% of the asymptomatic group scored the maximum value, thus, data were not normally distributed.

**Figure 1:**
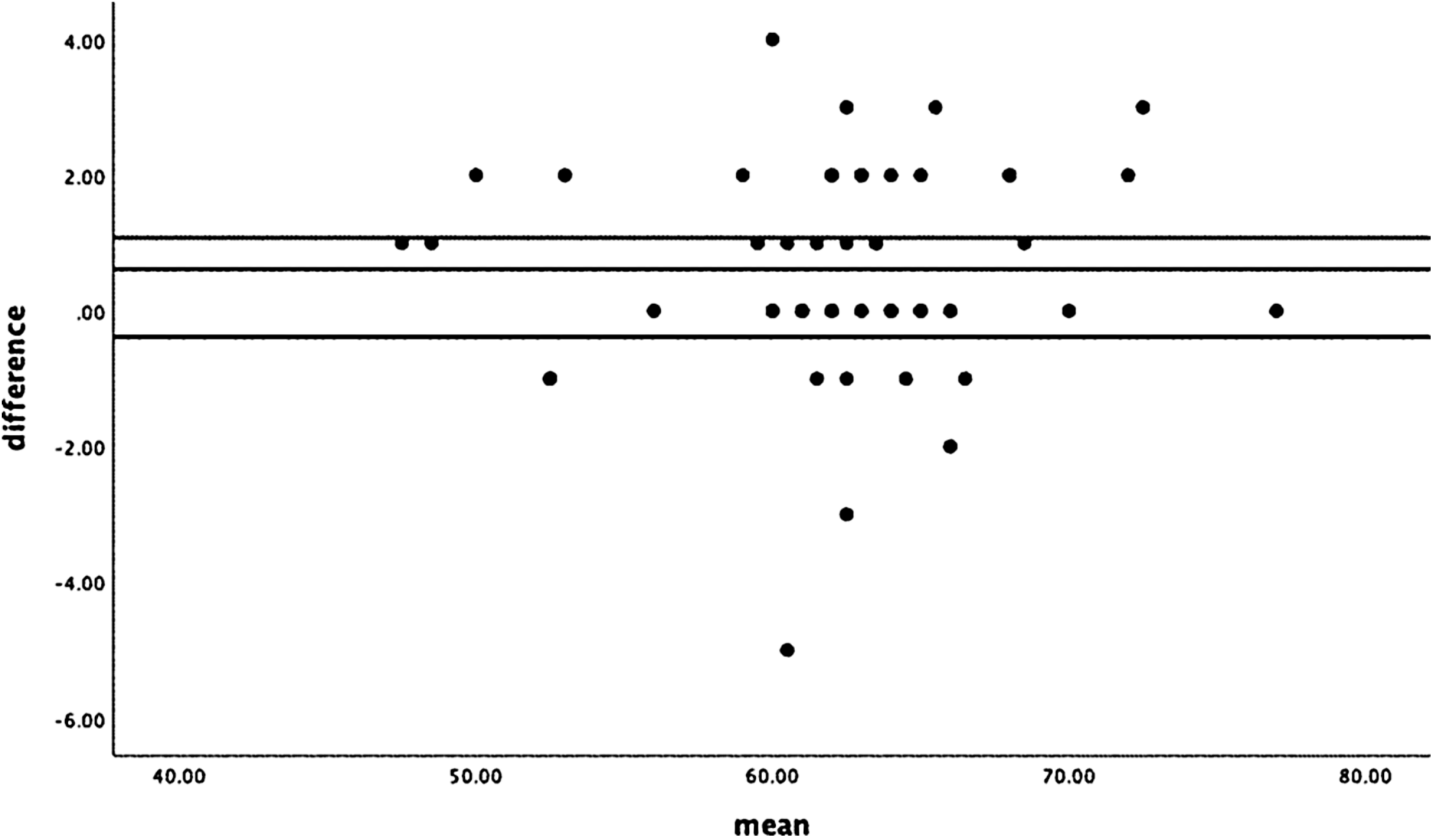
Bland–Altman plot.

Bland–Altman plots showed no significant or relevant differences from test to retest in the total score with mean differences below 1 (0.612) (Fig. 1).

## Discussion

The purpose of the current study was to translate the VISA-G (Fearon et al., 2015) from English into the Danish language (VISA-G.DK), to conduct a cross-cultural adaptation to a Danish context and to assess the VISA-G.DK reliability.

The VISA-G.DK has been shown to have an excellent internal consistency and a strong test–retest reliability. The SEM at a 95% confidence interval is shown to be ±1.2 points. This implies that ±1 SEM (68% confidence) of the ‘true’ score can be found between ±0.6 points from the observed score, and ±2 SEM (95% confidence) is between ±1.2 points of the observed score.

The MDC is ±3.17. Therefore, a change in the VISA-G.DK score of at least 3.17 points (on a scale of 0–100) would be considered to be a true change in the VISA-G.DK score, and not a potential result of measurement error. The Bland–Altman plots showed no significant from test to retest in the total score. No floor and ceiling effects were observed in the patient group, however in the asymptomatic group an expected ceiling effect was shown.

An internationally recommended translation procedure was used to produce a Danish version of the VISA-G questionnaire (Fearon et al., 2015). The rigorous translation methodology used ensured the questionnaire was translated and adapted into a Danish context. Cognitive interviews were used to provide a face validation of the translated versions of the VISA-G (Fearon et al., 2015), thereby minimizing non-responses and response errors (Wild et al., 2005). No major problems were observed in the forward and backward translations of the questionnaires.

The goal for test–retest reliability assessments is to distinguish true score differences from random and transitory measurement error (Polit, 2014). Studies of test–retest reliability for health-related QOL instruments have used varying intervals between test administrations, with an earlier study showing no statistically significant differences in test–retest reliability studies undertaken between 2 days and 2 weeks (Marx et al., 2003). We chose to use a 1 week between test and retest interval for practical reasons at the clinic involved.

The test–retest respondents were chosen within the population most often affected by GTPS (Fearon et al., 2014a; Barratt, Brookes & Newson, 2017). Recruiting respondents who were asymptomatic, ensured that the test–retest testing was based on the understanding of the questionnaire, thus further validating the translation. We also recruited symptomatic participants as this is the target population and fluctuating pain or functional responses between the 2-time intervals may cause validation and reliability scores to deflate (Tsang, Royse & Terkawi, 2017; Marx et al., 2003). In this study there were no major differences in reliability between the symptomatic and asymptomatic group. Function or pain, within the symptomatic group, would not be expected to vary to a large extent within the 1-week time span between test and retesting, thereby these factors are unlikely to have influenced the reliability.

The VISA-G.DK was found to have high levels of intra-rater reliability and internal consistency. These data are consistent with the English version of the VISA-G (Fearon et al., 2015) and the VISA-H (Cacchio, De Paulis & Maffulli, 2014). In addition, the VISA-G.DK like the English version (Fearon et al., 2015) is resistant to ceiling and floor effects, which is not the case with the English version of the mHHS or the Oswestry Disability Index, two other scores commonly used to assess GTPS and gluteal tendon tears (Ebert et al., 2019). Further, we are the first to report the MDC and the SEM for any version of the VISA-G. This means that clinicians who see a change of greater than 3 can be confident that this is a real change, not change due to measurement variation.

The testing of the VISA-G.DK was undertaken on a suitable sample size, meaning we have minimised the possibility of response bias in test–retests (Draugalis & Plaza, 2009). The sample size calculation suggested that a minimum of 49 respondents was needed for our primary analyses (Walter, Eliasziw & Donner, 1998; Polit, 2014; De Vet et al., 2011). The recruitment of 63 people per group and the high response rate, well above the minimum required (50–60%) (Draugalis & Plaza, 2009) meant we were well within the calculated 49 per group. The response rate of 78% the symptomatic group differs compared to the asymptomatic group; however the minimum number of respondents was met.

The Danish versions were completed within 1:20 and 8:24 min for the asymptomatic group of respondents and between 2:36 and 9:56 for the symptomatic group, thereby the VISA-G.DK seems feasible and field-friendly for investigating and monitoring GTPS pain and function. The test–retest study showed substantial agreement between test and retest in the non-symptomatic respondent group.

## Conclusion

The translated version of the VISA-G.DK questionnaire was linguistically and culturally equivalent to the original version. The translated score showed good reliability.

### Limitation

The 1-week period between test and retest may have induced recall bias, however, if this period was extended, pain and function scores may have changed due to other factors than cognitive understanding of the questionnaire. In an earlier study by Frohm et al. (2004) examining the VISA-P, the majority of participants (66%) were asymptomatic.

### Perspectives

Publishing the translation of a health measurement instrument is important to avoid the emergence of multiple versions of the instrument and to demonstrate that the translation procedure was rigorous. It also facilitates the comparison of findings between and within countries (Marshall et al., 2000). The translation of the VISA-G.DK to a Danish version was performed using rigorous methodology, adapted culturally, and face validated to a Danish context. Further research should be used to establish the psychometric properties of the VISA-G.DK in a clinical setting with a relevant sample of patients with established GTPS. An ongoing study (N-20180036. Clinical Predictors of Extracorporal Shockwave Therapy Efficacy in patients presenting with lateral hip pain) will endeavour to do this.

## Supplemental Information

10.7717/peerj.8724/supp-1 Supplemental Information 1 VISA-G.DK questionnaire in Danish.

10.7717/peerj.8724/supp-2 Supplemental Information 2 Guide for applying and scoring VISA-G.DK questionnaire in Danish.

10.7717/peerj.8724/supp-3 Supplemental Information 3 Raw data background for VISA-G.DK assessment.

10.7717/peerj.8724/supp-4 Supplemental Information 4 Instructions for implementation of the VISA-G.DK in Danish.

## Additional Information and Declarations

### Data Availability

The following information was supplied regarding data availability: The raw measurements are available in the Supplemental Files.
