# Supplementary material for: Translation, validation and test–retest reliability of the VISA-G patient-reported outcome tool into Danish (VISA-G.DK)
Source: PeerJ. 2020 Mar 4;8:e8724. doi: 10.7717/peerj.8724 (PMC7060749; doi:10.7717/peerj.8724)
Supplement: Supplemental Information 1 [file peerj-08-8724-s001.docx]

**VISA-G.DK Spørgeskema**

**Navn……………………………………………… Fødselsdag ………………………………………………..**

Sæt venligst kun kryds i én rubrik for hvert spørgsmål. Vælg den rubrik, der passer bedst til dig – det behøver ikke at passe fuldstændigt. Alle spørgsmålene er relateret til dine smerter i hoften.

**Spørgsmål 1: Mine sædvanlige gennemsnitlige hoftesmerter er…..**

| 0 | 1 | 2 | 3 | 4 | 5 | 6 | 7 | 8 | 9 | 10 |
| --- | --- | --- | --- | --- | --- | --- | --- | --- | --- | --- |

ingen smerte værst tænkelige smerte

**Spørgsmål 2: Jeg kan ligge på min ømme hofte**

☐ I længere end 1 time

☐ I 30 til 59 minutter, så må jeg flytte mig

☐ I 15 til 29 minutter, så må jeg flytte mig

☐ I 5 til 14 minutter, så må jeg flytte mig

☐ Jeg kan slet ikke ligge på min ømme side

**Spørgsmål 3: Gå op eller ned af en trappe**

☐ Jeg kan gå på trapper på normal vis uden hoftesmerter

☐ Jeg kan gå på trapper på normal vis med lette hoftesmerter

☐ Jeg kan gå på trapper på normal vis når jeg holder ved gelænderet på grund af mine hoftesmerter

☐ Jeg tager ét trin ad gangen på trappen og holder ved gelænderet på grund af mine hoftesmerter

☐ Jeg kan ikke gå på trapper overhovedet på grund af mine hoftesmerter

**Spørgsmål 4: Gå op eller ned af en rampe eller skråning**

☐ Jeg kan gå på normal vis op og ned af en rampe eller skråning uden hoftesmerter

☐ Jeg kan gå på normal vis op og ned af en rampe eller skråning med lette hoftesmerter

☐ Jeg har noget besvær med at gå op og ned af en rampe eller skråning på grund af mine hoftesmerter

☐ Jeg har store problemer med at gå op og ned af en rampe eller skråning på grund af mine hoftesmerter

☐ Jeg er ikke i stand til at gå op og ned af en rampe eller skråning på grund af mine hoftesmerter

**Spørgsmål 5: Efter at have siddet ned i 30 minutter, hvordan er dét at rejse sig til stående, for derefter at gå?**

☐ Ikke noget problem

☐ Besværligt de første par skridt

☐ Jeg er nødt til at stå stille et øjeblik eller to, før jeg går

☐ Jeg er nødt til at stå stille i mindre end 20 sekunder, før jeg går

☐ Jeg er nødt til at stå stille i mere end 20 sekunder, før jeg går

**Spørgsmål 6: Arbejde i hus og have (eller lignende aktivitet)**

☐ Jeg kan arbejde i mit hus og/eller have i en time eller mere

☐ På grund af mine hoftesmerter kan jeg kun arbejde i mit hus og/eller have i 30 til 60 minutters intervaller

☐ På grund af mine hoftesmerter udfører jeg begrænset arbejde i mit hus og have

☐ På grund af mine hoftesmerter udfører jeg kun meget begrænset arbejde i mit hus, og jeg arbejder ikke i haven

☐ På grund af mine hoftesmerter udfører jeg ikke arbejde i mit hus eller have

**Spørgsmål 7: Deltager du for øjeblikket i regelmæssig motion, fysisk aktivitet eller sport?**

☐ Ja – jeg kan dyrke motion, som jeg plejer

☐ Noget mindre end jeg plejer

☐ Betydeligt mindre end jeg plejer

☐ Nej – Jeg er ikke i stand til at dyrke motion, jeg vil ikke eller jeg har ikke tid

**Spørgsmål 8 indeholder tre sektioner. *Besvar venligst KUN enten sektion A, B eller C.***

**Sektion A: Hvis du kan svare ja til følgende: Mine hoftesmerter er så stærke, at det afholder mig fra at gå, shoppe, løbe eller udføre anden vægtbærende motion fx gang, shopping, løb, knæbøjning (”squats”) eller fremfald (”lunges”)? , skal du besvare sektion A. Hvis du svare nej, fortsæt til sektion B eller C.**

**Hvor meget af denne form for aktivitet udfører du så hver dag?**

☐ Jeg laver ikke ekstra aktivitet for benene – Jeg bevæger mig kun rundt i huset

☐ Jeg er aktiv mindre end 10 minutter

☐ Jeg er aktiv 10-19 minutter

☐ Jeg er aktiv 20-29 minutter

☐ Jeg er aktiv mere end 30 minutter

**Sektion B: Hvis du kan svare ja til følgende: Jeg har hoftesmerter i forbindelse med motion, men smerterne afholder mig ikke fra at gå, shoppe, løbe eller udføre andre typer af vægtbærende motion fx gang, shopping, løb, knæbøjning (”squats”) eller fremfald (”lunges”)?, skal du besvare sektion B. Hvis du svare nej, fortsæt til sektion C.**

**Hvor meget af denne form for aktivitet udfører du så hver dag**

☐ Jeg laver ikke ekstra aktivitet for benene – Jeg bevæger mig kun rundt i huset

☐ Jeg er aktiv mindre end 10 minutter

☐ Jeg er aktiv 10-19 minutter

☐ Jeg er aktiv 20-29 minutter

☐ Jeg er aktiv mere end 30 minutter

**C: Hvis du kan svare ja til følgende: Du har ikke hoftesmerter, når du går, shopper, løber eller udfører andre typer af vægtbærende motion, fx gang, shopping, løb, knæbøjning (”squats”) eller fremfald (”lunges”)?, skal du besvare sektion C**

**Hvor meget af denne form for aktivitet udfører du så hver dag**

☐ Jeg laver ikke ekstra aktivitet for benene – Jeg bevæger mig kun rundt i huset

☐ Jeg er aktiv mindre end 10 minutter

☐ Jeg er aktiv 10-19 minutter

☐ Jeg er aktiv 20-29 minutter

☐ Jeg er aktiv mere end 30 minutter

**TOTAL SCORE = /100**
