# Supplementary material for: Translation, validation and test–retest reliability of the VISA-G patient-reported outcome tool into Danish (VISA-G.DK)
Source: PeerJ. 2020 Mar 4;8:e8724. doi: 10.7717/peerj.8724 (PMC7060749; doi:10.7717/peerj.8724)
Supplement: Supplemental Information 4 [file peerj-08-8724-s004.docx]

# vejledning Visa-G.DK

Udvikling af VISA-G ^(1).^

Visa-G spørgeskemaet er udviklet med henblik på at evaluere, hvor alvorligt en person er påvirket af gluteal tendinopati. Skemaet er udviklet og testet med det formål, at det skal benyttes i sin helhed, selvom enkelte sektioner vil kunne være af interesse for klinikeren i forhold til at monitorere personens udvikling.

Minimal Clinical Difference (MCD) er i skrivende stund ikke færdigberegnet, men det forventes at forandring vil kunne registreres i løbet af uger snarere end dage.

Den kliniske diagnose dette spørgeskema er anvendelig til er ^(2)^:

- Smerter på ydersiden af hoften over trochanter major
- Smerter ved palpation af trochanter major
- Reproducerbare smerter ved udførelse af FABER testen
- Ingen problemer med påtagning af sko eller strømper

Scoren er valid for personer med samtidige lænderygsmerter. Spørgeskemaet har ikke været testet i forhold til personer med samtidig hofteartrose. Hvis FABER testen reproducerer lyskesmerte, har personen muligvis en intraartikulær problematik ^(2).^

## Anvendelse af spørgeskemaet:

Når spørgeskemaet skal udfyldes første gang, anbefales det at forklare patienten hvert enkelt spørgsmål grundigt.

Spørgsmål 8 er særligt vigtigt, idet dette angiver graden af funktionsniveau, og kan give anledning til forvirring, da dette spørgsmål består af 3 sektioner A, B og C.

**Patienten skal kun udfylde enten sektion A, B eller C.** Hvilken del den enkelte patient skal udfylde, afhænger af patientens nuværende smerteniveau (se beskrivelsen af de enkelte sektioner i spørgeskemaet). Være sikker på at patienten forstår forskellen mellem de enkelte sektioner.

Et eksempel: En patient der ikke har smerter når vedkommende går, shopper eller udfører andre typer af vægtbærende motion score: (Sektion C, score=6).

Et andet eksempel: En patient har hoftesmerter i forbindelse med motion, men smerterne afholder ikke vedkommende fra at gå, shoppe, løbe eller udføre andre typer af vægtbærende motion. Vedkommende kan dog kun gennemføre aktiviteten i 20-29 minutter. Så vil vedkommende score: (Sektion B, score=15).

Når alle spørgsmål er besvaret, udregnes den samlede score ud af 100 mulige point (Se spørgeskema med score.)

- Hvis patienten undlader at svare på et spørgsmål (bør undgås), udregnes scoren ud fra de mulige antal point. Dette er klinisk anvendeligt, men dette er ikke testet og valideret.
- Hvis patienten alligevel besvarer alle tre sektioner i spørgsmål 8, anvendes besvarelsen af lavest muligt scenarie = sektion A.

Kilder:

1. Fearon AM, Ganderton C, Scarvell JM, Smith PN, Neeman T, Nash C, et al. Development and validation of a VISA tendinopathy questionnaire for greater trochanteric pain syndrome, the VISA-G. Man Ther. 2015;20(6):805-13.

2. Fearon AM, Scarvell JM, Neeman T, Cook JL, Cormick W, Smith PN. Greater trochanteric pain syndrome: defining the clinical syndrome. Br J Sports Med. 2013;47(10):649-53.

**VISA-G.DK Spørgeskema med score**

**Navn……………………………………………… Fødselsdag ………………………………………………..**

Sæt venligst kun kryds i én rubrik for hvert spørgsmål. Vælg den rubrik, der passer bedst til dig – det behøver ikke at passe fuldstændigt. Alle spørgsmålene er relateret til dine smerter i hoften.

**Spørgsmål 1: Mine sædvanlige gennemsnitlige hoftesmerter er….. (f.eks.: tallet 0 = scorer 10 point, tallet 6 scorer 4 point, tallet 10 scorer 0 point)**

| 0 10 | 1 9 | 2 8 | 3 7 | 4 6 | 5 5 | 6 4 | 7 3 | 8 2 | 9 1 | 10 0 |
| --- | --- | --- | --- | --- | --- | --- | --- | --- | --- | --- |

ingen smerte værst tænkelige smerte

**Spørgsmål 2: Jeg kan ligge på min ømme hofte**

10 ☐ I længere end 1 time

7 ☐ I 30 til 59 minutter, så må jeg flytte mig

5 ☐ I 15 til 29 minutter, så må jeg flytte mig

2 ☐ I 5 til 14 minutter, så må jeg flytte mig

0 ☐ Jeg kan slet ikke ligge på min ømme side

**Spørgsmål 3: Gå op eller ned af en trappe**

10 ☐ Jeg kan gå på trapper på normal vis uden hoftesmerter

7 ☐ Jeg kan gå på trapper på normal vis med lette hoftesmerter

5 ☐ Jeg kan gå på trapper på normal vis, når jeg holder ved gelænderet på grund af mine hoftesmerter

2 ☐ Jeg tager ét trin ad gangen på trappen og holder ved gelænderet på grund af mine hoftesmerter

0 ☐ Jeg kan ikke gå på trapper overhovedet på grund af mine hoftesmerter

**Spørgsmål 4: Gå op eller ned af en rampe eller skråning**

10☐ Jeg kan gå på normal vis op og ned af en rampe eller skråning uden hoftesmerter

7 ☐ Jeg kan gå på normal vis op og ned af en rampe eller skråning med lette hoftesmerter

5 ☐ Jeg har noget besvær med at gå op og ned af en rampe eller skråning på grund af mine hoftesmerter

2 ☐ Jeg har store problemer med at gå op og ned af en rampe eller skråning på grund af mine hoftesmerter

0 ☐ Jeg er ikke i stand til at gå op og ned ad en rampe eller skråning på grund af mine hoftesmerter

**Spørgsmål 5: Efter at have siddet ned i 30 minutter, hvordan er dét at rejse sig til stående, for derefter at gå?**

10 ☐ Ikke noget problem

7 ☐ Besværligt de første par skridt

5 ☐ Jeg er nødt til at stå stille et øjeblik eller to, før jeg går

2 ☐ Jeg er nødt til at stå stille i mindre end 20 sekunder, før jeg går

0 ☐ Jeg er nødt til at stå stille i mere end 20 sekunder, før jeg går

**Spørgsmål 6: Arbejde i hus og have (eller lignende aktivitet)**

10 ☐ Jeg kan arbejde i mit hus og/eller have i en time eller mere

7 ☐ På grund af mine hoftesmerter kan jeg kun arbejde i mit hus og/eller have i 30 til 59 minutters intervaller

5 ☐ På grund af mine hoftesmerter udfører jeg begrænset arbejde i mit hus og have

2 ☐ På grund af mine hoftesmerter udfører jeg kun meget begrænset arbejde i mit hus, og jeg arbejder ikke i haven

0 ☐ På grund af mine hoftesmerter udfører jeg ikke arbejde i mit hus eller have

**Spørgsmål 7: Deltager du for øjeblikket i regelmæssig motion, fysisk aktivitet eller sport?**

10 ☐ Ja – jeg kan dyrke motion, som jeg plejer

7 ☐ Noget mindre end jeg plejer

4 ☐ Betydeligt mindre end jeg plejer

0 ☐ Nej – Jeg er ikke i stand til at dyrke motion, jeg vil ikke eller jeg har ikke tid

**Spørgsmål 8 indeholder tre sektioner. *Besvar venligst KUN enten sektion A, B eller C.***

**Sektion A: Hvis du kan svare ja til følgende: Mine hoftesmerter er så stærke, at det afholder mig fra at gå, shoppe, løbe eller udføre anden vægtbærende motion fx gang, shopping, løb, knæbøjning (”squats”) eller fremfald (”lunges”)? , skal du besvare sektion A. Hvis du svare nej, fortsæt til sektion B eller C.**

**Hvor meget af denne form for aktivitet udfører du så hver dag?**

0 ☐ Jeg laver ikke ekstra aktivitet for benene – Jeg bevæger mig kun rundt i huset

2 ☐ Jeg er aktiv mindre end 10 minutter

5 ☐ Jeg er aktiv 10-19 minutter

7 ☐ Jeg er aktiv 20-29 minutter

10 ☐ Jeg er aktiv mere end 30 minutter

**Sektion B: Hvis du kan svare ja til følgende: Jeg har hoftesmerter i forbindelse med motion, men smerterne afholder mig ikke fra at gå, shoppe, løbe eller udføre andre typer af vægtbærende motion fx gang, shopping, løb, knæbøjning (”squats”) eller fremfald (”lunges”)?, skal du besvare sektion B. Hvis du svare nej, fortsæt til sektion C.**

**Hvor meget af denne form for aktivitet udfører du så hver dag**

0 ☐ Jeg laver ikke ekstra aktivitet for benene – Jeg bevæger mig kun rundt i huset

5 ☐ Jeg er aktiv mindre end 10 minutter

10 ☐ Jeg er aktiv 10-19 minutter

15 ☐ Jeg er aktiv 20-29 minutter

20 ☐ Jeg er aktiv mere end 30 minutter

**C: Hvis du kan svare ja til følgende: Du har ikke hoftesmerter, når du går, shopper, løber eller udfører andre typer af vægtbærende motion, fx gang, shopping, løb, knæbøjning (”squats”) eller fremfald (”lunges”)?, skal du besvare sektion C**

**Hvor meget af denne form for aktivitet udfører du så hver dag**

6 ☐ Jeg laver ikke ekstra aktivitet for benene – Jeg bevæger mig kun rundt i huset

12 ☐ Jeg er aktiv mindre end 10 minutter

18 ☐ Jeg er aktiv 10-19 minutter

24 ☐ Jeg er aktiv 20-29 minutter

30 ☐ Jeg er aktiv mere end 30 minutter

**TOTAL SCORE = /100**
